# HIV-Associated Histoplasmosis Early Mortality and Incidence Trends: From Neglect to Priority

**DOI:** 10.1371/journal.pntd.0003100

**Published:** 2014-08-21

**Authors:** Antoine Adenis, Mathieu Nacher, Matthieu Hanf, Vincent Vantilcke, Rachida Boukhari, Denis Blachet, Magalie Demar, Christine Aznar, Bernard Carme, Pierre Couppie

**Affiliations:** 1 Inserm CIC 1424, Centre d'Investigation Clinique Antilles Guyane, Centre Hospitalier de Cayenne, Cayenne, France; 2 Equipe EA3593, Epidémiologie des Parasitoses et des Mycoses Tropicales, Université des Antilles et de la Guyane, Cayenne, France; 3 Service de Médecine Interne, Centre Hospitalier de l'Ouest Guyanais, Saint Laurent du Maroni, France; 4 Laboratoire de Biologie Médicale, Centre Hospitalier de l'Ouest Guyanais, Saint Laurent du Maroni, France; 5 Laboratoire Hospitalo-Universitaire de Parasitologie-Mycologie, Centre Hospitalier de Cayenne, Cayenne, France; 6 Unité des Maladies Infectieuses et Tropicales, Centre Hospitalier de Cayenne, Cayenne, France; 7 Service de Dermatologie Vénérologie, Centre Hospitalier de Cayenne, Cayenne, France; Baylor College of Medicine, United States of America

## Abstract

**Background:**

Histoplasmosis is an endemic fungal infection in French Guiana. It is the most common AIDS-defining illness and the leading cause of AIDS-related deaths. Diagnosis is difficult, but in the past 2 decades, it has improved in this French overseas territory which offers an interesting model of Amazonian pathogen ecology. The objectives of the present study were to describe the temporal trends of incidence and mortality indicators for HIV-associated histoplasmosis in French Guiana.

**Methods:**

A retrospective study was conducted to describe early mortality rates observed in persons diagnosed with incident cases of HIV-associated *Histoplasma capsulatum* var. *capsulatum* histoplasmosis admitted in one of the three main hospitals in French Guiana between 1992 and 2011. Early mortality was defined by death occurring within 30 days after antifungal treatment initiation. Data were collected on standardized case report forms and analysed using standard statistical methods.

**Results:**

There were 124 deaths (45.3%) and 46 early deaths (16.8%) among 274 patients. Three time periods of particular interest were identified: 1992–1997, 1998–2004 and 2005–2011. The two main temporal trends were: the proportion of early deaths among annual incident histoplasmosis cases significantly declined four fold (χ2, *p*<0.0001) and the number of annual incident histoplasmosis cases increased three fold between 1992–1997 and 1998–2004, and subsequently stabilized.

**Conclusion:**

From an occasional exotic diagnosis, AIDS-related histoplasmosis became the top AIDS-defining event in French Guiana. This was accompanied by a spectacular decrease of early mortality related to histoplasmosis, consistent with North American reference center mortality rates. The present example testifies that rapid progress could be at reach if awareness increases and leads to clinical and laboratory capacity building in order to diagnose and treat this curable disease.

## Author Summary

Histoplasmosis is an endemic fungal infection in French Guiana. It is the most common AIDS-defining illness and the leading cause of AIDS-related deaths. Diagnosis is difficult, but in the past 2 decades, it has improved. The objectives of the present study were to describe the temporal trends of incidence and mortality indicators for HIV-associated histoplasmosis in French Guiana. A retrospective study was conducted to describe early mortality rates observed in persons diagnosed with incident cases of HIV-associated histoplasmosis admitted in one of the three main hospitals of French Guiana between 1992 and 2011. Early mortality was defined by death occurring within 30 days after antifungal treatment initiation. Data were collected on standardized case report forms and analysed using standard statistical methods. Among 274 patients there were 46 early deaths (16.8%). The two main temporal trends were: the proportion of early deaths significantly divided four fold and the number of annual incident histoplasmosis cases increased three fold. The present example testifies that rapid progress could be at reach if awareness increases and leads to clinical and laboratory capacity building in order to diagnose and treat this curable disease.

## Introduction

French Guiana is a French overseas territory, located in the North-Eastern part of South America. The Human Immunodeficiency Virus (HIV) epidemic there is the most preoccupying among French territories [1]. During the Highly Active AntiRetroviral Therapy (HAART) era, disseminated histoplasmosis has remained the most common Acquired Immunodeficiency Syndrome (AIDS) defining illness with an incidence of 15.4/1000 person-years in HIV-infected patients [2].

In immunocompetent patients, *Histoplasma capsulatum* var. *capsulatum* infection is typically asymptomatic or pauci-symptomatic and spontaneous resolution is the rule in the great majority of cases [3]. On the contrary, in HIV-infected patients it presents mostly as a disseminated infection. With the worsening of the immunosuppression, the disease progression is often rapid and always fatal in the absence of treatment [4].

Thus, different studies have observed up to 39% of deaths following diagnosis in endemic areas, where it is supposedly well known, and 58% in non endemic areas, where it is perhaps less known [5], [6]. In endemic areas, although there are different outcome measures and inclusion criteria, the death rates observed in AIDS-associated histoplasmosis differ between the USA (12–23%) and South America (19–39%) [6]. Hypotheses advanced to explain these differences are a delayed recognition due to the lack of awareness of physicians, a delayed diagnosis due to the lack of diagnostic facilities and the late presentation of HIV-infected patients in resource limited settings [6], [7], [8]. Delayed treatment due to the unavailability of the most effective therapy in severe cases, the impossibility of monitoring drug concentrations and/or drug-drug interactions with antituberculosis treatments are other possible explanations [6].

In French Guiana, disseminated histoplasmosis has also been the leading cause of death among HIV-infected patients [9]. Despite HIV care and treatment standards close to those in Mainland France, the mortality rate of AIDS-associated histoplasmosis remains high in the HAART era (30.7% at 6 months and 17.5 at 1 month), whereas in Mainland France, a non-endemic area, this mortality rate was divided by two [10], [11].

The objective of this study was to describe the temporal trends of incidence and mortality indicators for AIDS-associated histoplasmosis in French Guiana. This knowledge is important to guide and improve AIDS-associated histoplasmosis diagnosis, care and treatment, and to illustrate that awareness and standard practices in mycology can dramatically change prognosis.

## Materials and Methods

### Ethics Statement

Since 1992, an anonymized database compiles retrospectively and continuously *Histoplasma capsulatum var. capsulatum* histoplasmosis confirmed incident cases diagnosed in HIV-infected patients according to the case definition of the European Organization for Research and Treatment of Cancer/Invasive Fungal Infections Cooperative Group and the National Institute of Allergy and Infectious Diseases Mycoses Study Group (EORTC/MSG) Consensus Group [12]. The revised EORTC/MSG criteria defining a proven case of histoplasmosis are: recovery in culture from a specimen obtained from the affected site or from blood; and/or histopathologic or direct microscopic demonstration of appropriate morphologic forms with a truly distinctive appearance characteristic such as intracellular yeasts forms in a phagocyte in a peripheral blood smear or in tissue macrophages. By contrast, molecular methods of detecting fungi in clinical specimens, such as Polymerase Chain Reaction (PCR), were not included in the classifications of “proven,” “probable,” and “possible” invasive fungal disease (IFD) definitions because there is as yet no standard, and none of the techniques has been clinically validated.

All HIV-infected patients hospitalized or seen in the outpatient department before admission, suspicious for histoplasmosis and receiving antifungal therapy in one of the three main hospitals of French Guiana (the Centre Hospitalier de Cayenne (CHC), the Centre Hospitalier Médico-Chirurgical de Kourou (CMCK) and the Centre Hospitalier de l'Ouest Guyanais in Saint Laurent du Maroni (CHOG), were identified and checked for a confirmed diagnosis of histoplasmosis in all laboratories where biological samples were sent. Then, they were finally enrolled according to the following inclusion criteria: age >18 years, admission in one of the three hospitals (the inclusion date corresponding to the date of antifungal treatment initiation), confirmed HIV infection (by Western blot), confirmed incident histoplasmosis infection (EORTC/MSG criteria), and baseline blood screening within 7 days prior to antifungal therapy initiation. Non inclusion criteria were: histoplasmosis relapse or diagnosis of histoplasmosis relying only on Histoplasma Polymerase Chain Reaction (PCR). Data were collected on a standardized form and included sociodemographic, clinical, biologic, radiologic, therapeutic and survival information. These data were then entered in an anonymized database. Ethical approval was obtained for the database and related studies (IRB0000388, FWA00005831). A descriptive study of the patients included in this database until April 2007 was published elsewhere [10].

### Methods

An observational, retrospective and multicentric study was conducted from 01/01/1992 to 09/30/2011, using the French Guiana HIV-Histoplasmosis database described above.

In this study, the primary endpoint was the vital status on day 30 following antifungal therapy initiation. Patients lost to follow up within 30 days following antifungal therapy initiation, or deceased with an unknown date of death, or presenting a relapse of histoplasmosis were excluded from the analysis.

This early death criterion appeared as a good compromise to attribute mortality to the histoplasmosis infectious episode under consideration, in a context of severe immunosuppression favouring multiple opportunistic pathogens, ensuring simplicity and reproducibility of the study.

The statistical analysis was performed using STATA 10.0 (College Station, Texas, USA) (38). Descriptive analysis used proportions, medians and trend χ^2^ test.

## Results

There were 278 patients with AIDS-associated histoplasmosis. Four cases were excluded before the analysis (3 because they were lost to follow up and one because of an unknown date of death). Their socio-demographic characteristics and median CD4 count did not differ from the 274 patients finally selected in this study (data not shown).

Among the 274 patients selected for whom the vital status at 30 days after antifungal therapy initiation was known, there were 124 deaths (45.3%). The median time to death was 110 days (Interquartile Range [IQR] = 13–481) and the median age at the time of death was 39 years (IQR = 33–47). Early death occurred in 46 patients (16.8%) with a median survival time of 7 days (IQR = 3–16) after antifungal treatment initiation. The median age at the time of early death was 37 years (IQR = 32–47).

**Figure 1:**
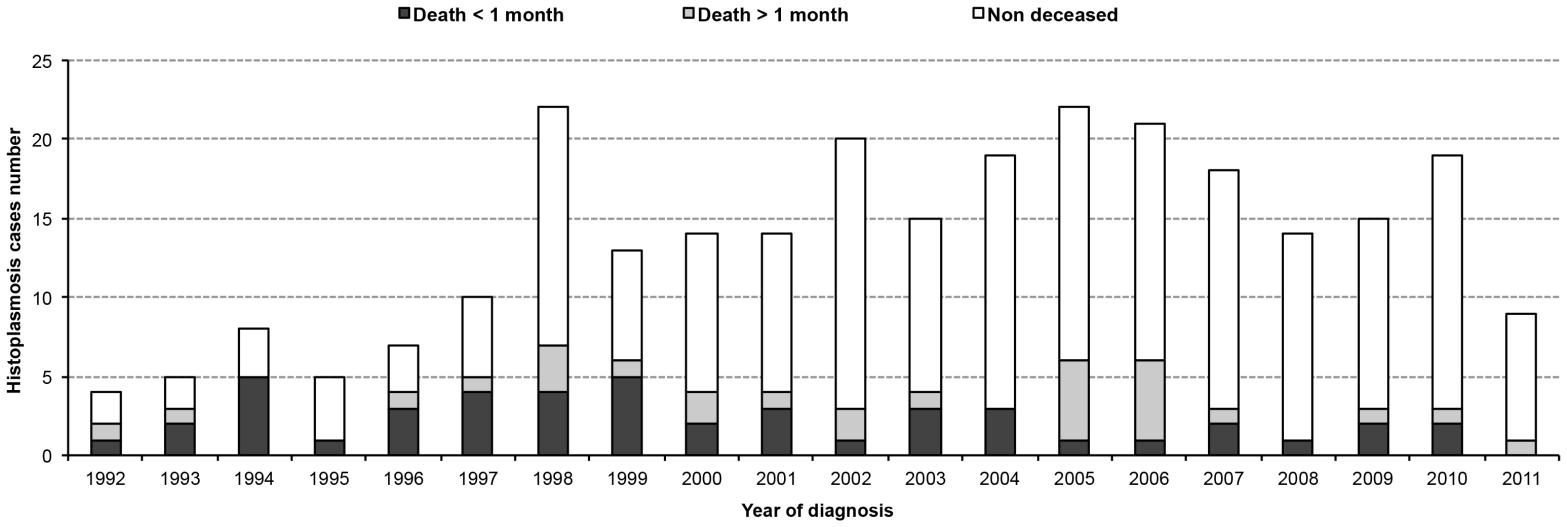
Number of deaths and early deaths observed among annual incident histoplasmosis cases diagnosed in the three main hospitals of French Guiana between 01/01/1992 and 09/30/2011.

Figure 1 shows that the proportion of deaths occurring the same year as the diagnosis of incident histoplasmosis cases remained stable around 5 deaths per year until 2005/2006 and then stabilized around 3 deaths per year. Among these deaths cases, almost half were early deaths until 2004. From 2005 onwards there was a notable decline of early deaths along with the overall decline of mortality. In addition, starting in 1998, the number of histoplasmosis cases diagnoses increased, and subsequently the number of incident cases oscillated between 14 and 22 cases per year. Data were incomplete for 2011, the study considering cases only until 09/30/2011.

**Figure 2:**
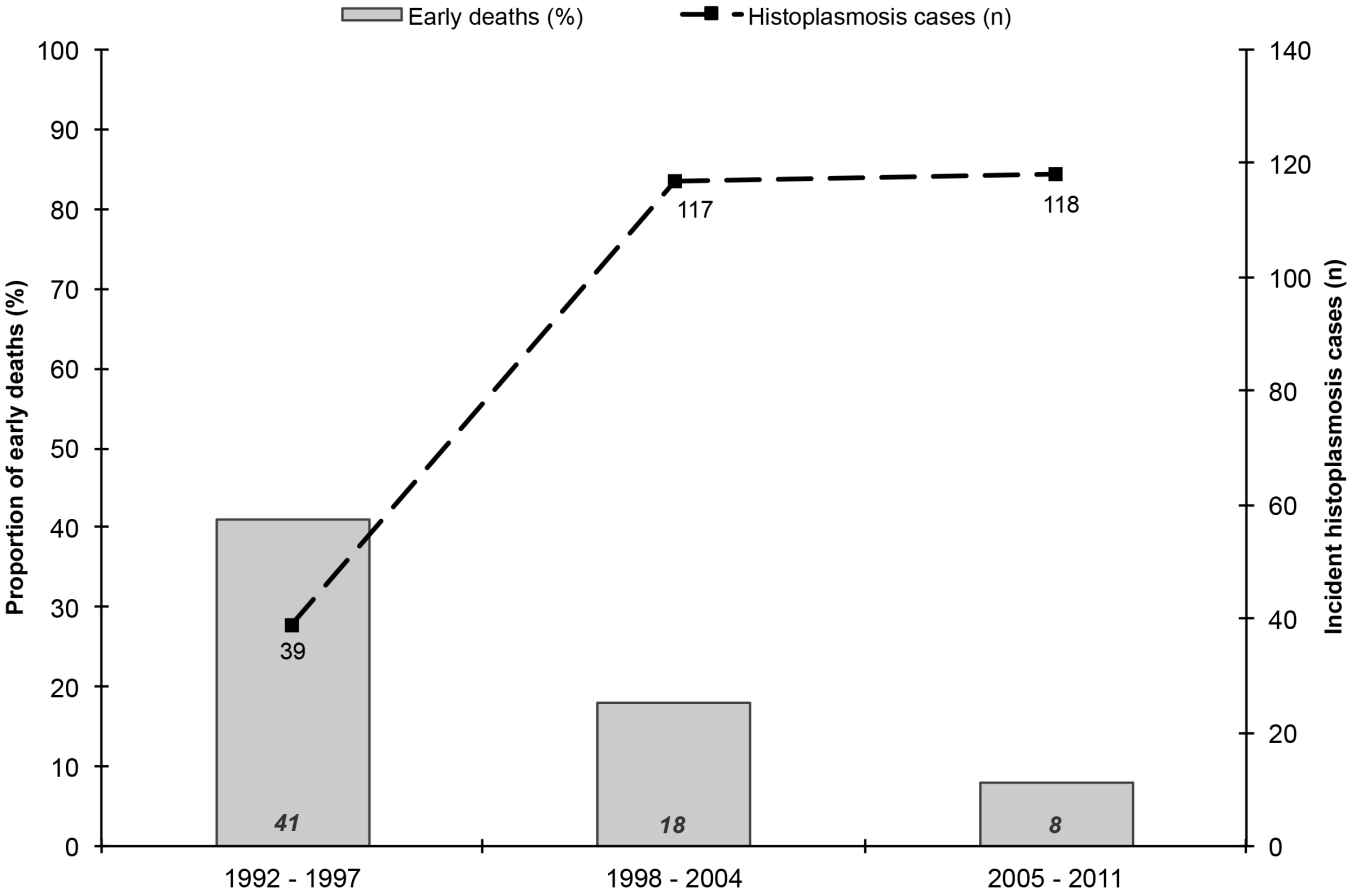
Incident histoplasmosis cases (n) and proportion of early deaths (%) observed in the three main hospitals of French Guiana between 01/01/1992 and 09/30/2011.

Thus, three time periods of particular interest have been identified: 1992–1997, 1998–2004 and 2005–2011. Figure 2 summarizes the two main temporal trends observed in Figure 1. First, the proportion of early deaths among annual incident histoplasmosis cases was significantly divided four fold (χ^2^, *p*<0.0001). Second, the number of annual incident histoplasmosis cases increased three fold between 1992–1997 and 1998–2004, and subsequently stabilized at the same level.

Table 1 showed that early deaths associated with histoplasmosis occurred mainly in men, late presenters with HIV infection (CD4 count <50/mm3) among whom 10% were on HAART on admission. The incident histoplasmosis cases were mainly disseminated and often recognized as the first AIDS-defining illness in the course of HIV infection. Fungal culture and direct examination were the main methods used for the diagnosis of histoplasmosis cases. The Real Time Polymerase Chain Reaction (RT-PCR) detection method for *Histoplasma* only became available during the 2005–2011 period. Amphotericin B and itraconazole were the first line antifungal regimen used to treat these patients. During the study period, liposomal amphotericin B and itraconazole became the standard antifungal regimen over deoxycholate amphotericin B and fluconazole, respectively.

**Table 1:** Description of baseline HIV infection and histoplasmosis infection characteristics and treatments in patients with AIDS-related histoplasmosis incident cases early death, in French Guiana, between 01/01/1992 and 09/30/2011.

	Study time period			
	1992–1997	1998–2004	2005–2011	Overall
	n = 16	n = 21	n = 9	n = 46
**Demographics and HIV characteristics on admission**				
Sex male, *n*(%)	11 (69)	14 (67)	5 (56)	30 (65)
Mean age +/− SD, years	38 (8)	37 (13)	44 (7)	39 (11)
HIV diagnosis <1 year, *n*/N(%)	1/4 (25)	4/7 (57)	6/9 (67)	11/20 (55)
Histoplasmosis as the first AIDS-defining illness, *n*(%)	11 (69)	15 (71)	8 (89)	34 (74)
Concomitant opportunistic infection, *n*(%)	7 (44)	8 (38)	3 (33)	18 (19)
Patient on HAART, *n*(%)	0 (0)	2 (10)	1 (11)	3 (7)
Median CD4 count (IQR 25–75%),/mm3*	15 (5–30)	43 (8–54)	33 (15–52)	24 (7–50)
**Histoplasmosis infection disease Classification**				
Progressive disseminated histoplasmosis, *n*(%)	14 (87)	20 (95)	9 (100)	43 (93)
Pulmonary histoplasmosis, *n*(%)	2 (13)	1 (5)	0 (0)	3 (7)
**Histoplasmsosis infection diagnostic methods** †				
Fungal culture, *n*/N (%)	8/15 (53)	19/20 (95)	8/9 (89)	35/44 (80)
Direct examination (MGG), *n*/N (%)	13/16 (81)	16/21 (76)	6/9 (67)	35/46 (76)
Pathology (PAS and silver staining), n/N (%)	7/10 (70)	4/6 (67)	1/1 (100)	12/17 (71)
RT-PCR, *n*/N (%)	0/0 (0)	0/0 (0)	4/4 (100)	4/4 (100)
Serology (Immunodiffusion), *n*/N (%)	0/0 (0)	0/1 (0)	0/1 (0)	0/2 (0)
**First-line antifungal regimen for histoplasmosis** ‡				
Deoxycholate amphotericin B (IV), *n*(%)	10 (63)	9 (43)	0 (0)	19 (41)
Itraconazole (oral), *n*(%)	4 (25)	9 (43)	5 (56)	18 (39)
Liposomal amphotericin B (IV), *n*(%)	0 (0)	4 (19)	5 (56)	9 (20)
Fluconazole (oral or IV), *n*(%)	2 (12)	0 (0)	1 (11)	3 (7)

* One CD4 count missing value during the 1992–1997 period.

† Good practices for fungal culture and serology were implemented in 1997–1998 and RT-PCR (Polymerase Chain Reaction using a Real-Time detection method) was implemented in 2006 in Cayenne General Hospital.

‡ 3 patients received amphotericin B (liposomal or deoxycholate) and itraconazole or fluconazole simultaneously.

SD: Standard Deviation, IQR 25–75%: Interquartile range 25%–75%, HIV: Human Immunodeficiency Virus, HAART: Highly Active Antiretroviral Therapy, MGG: May Grünwald Giemsa, IV: Intravenously.

## Discussion

This study described 19 years of experience in French Guiana. Three periods of interest and two main trends could be observed from 1998 onwards: the spectacular decrease of early deaths among incident histoplasmosis cases, and a simultaneous marked increase of the annual incidence of histoplasmosis cases. Whereas, during the same period, HIV prevalence in pregnant women was quite stable >1% since the 1990's: 0.8%–1.4% between 1992–1997, 1.2%–1.4% between 1998–2004 and 1.0%–1.2% between 2005–2011 [1], [13].

The increased number of annual histoplasmosis cases can be attributed to the development of medical mycology skills in hospitals laboratories, notably a reference university laboratory specialized in parasitology-mycology established since 1997 in Cayenne Hospital. By the same time, highly active antiretroviral therapy was introduced, which could have led to more patent cases of histoplasmosis due to the immune reconstitution inflammatory syndrome [14]. In addition, a PCR diagnostic method became available for histoplasmosis in 2006 [15]. Unfortunately, urinary antigen detection for histoplasmosis is still unavailable in French Guiana.

The sharp decline of the proportion of early deaths can be attributed to the improvement of the diagnostic capacity along with the improvement of the clinical management of HIV-infected patients following French recommendations [16]. Thus, French Guiana reached HIV-virological suppression levels comparable to those in Mainland France by 2004. In addition, this trend can also be attributed to the improvement of the clinical management of AIDS-related disseminated histoplasmosis cases. The accurate recognition of severe cases and the supply of liposomal amphotericin B since 1998, an effective and less nephrotoxic treatment recommended for severe disseminated histoplasmosis cases, were two important factors behind the progress.

This study had limitations. Data were collected retrospectively, which might have led to selection biases. Determining retrospectively if death was related to AIDS-associated histoplasmosis incident cases under study is challenging, considering the high percentage of concomitant opportunistic infections. Thus, we chose early death as the primary outcome because we thought that retrospectively it was the simplest and most reproducible indicator of histoplasmosis AIDS-related deaths.

Despite its limitations, this study showed that capacity building both in laboratory and clinical practice, effective drug availability both for HIV and histoplasmosis infections, and an effective bench to bed collaboration between actors progressively helped in reducing the burden of overall deaths and early deaths. Mortality indicators are now consistent with those described in North America, where the most effective and non invasive histoplasmosis diagnostic method is available. To further reduce early mortality, reducing diagnostic delays and antifungal therapy initiation is still a major objective. To reach it, a diagnostic method that meets the World Healh Organization's A.S.S.U.R.E.D. (Affordable, Sensitive, Specific, User-friendly, Rapid/Robust, Equipment-free and Delivered) should be developed.

Although our results may seem parochial, they illustrate the rapid progress that took place within a decade. The increased awareness of clinicians, who became more aggressive in their investigations, and the increased laboratory capacity led to find and treat a disease that was present but probably not identified and not treated in time. Thus, histoplasmosis, previously known as a mild disease in immunocompetent individuals, became a public health problem in HIV-infected patients, known by almost all health practitioners in French Guiana. By dealing with the mycology diagnostic tool box limitations and starting prompt presumptive antifungal treatment in HIV-infected patients it was possible to reduce early deaths considerably.

The historical 40% of early deaths observed in French Guiana, where histoplasmosis was known, plausibly reflects a low estimate of what happens in the Amazon region and probably beyond, where histoplasmosis is endemic but probably still widely misdiagnosed for tuberculosis and/or neglected [17]. Although cost effective strategies to prevent the disease and very effective diagnostic methods have been developed and are well known by scattered medical teams in Latin America [18], this knowledge does not percolate to too many HIV care units and hospital laboratories [19].

The present example testifies that rapid progress could be at reach if awareness increased and led to implement clinical and laboratory capacity building in order to diagnose and treat this curable disease before it is too late.
